# Digital Twin Smart City: Integrating IFC and CityGML with Semantic Graph for Advanced 3D City Model Visualization

**DOI:** 10.3390/s24123761

**Published:** 2024-06-09

**Authors:** Phuoc-Dat Lam, Bon-Hyon Gu, Hoang-Khanh Lam, Soo-Yol Ok, Suk-Hwan Lee

**Affiliations:** Department of Computer Engineering, Dong-A University, Busan 49315, Republic of Korea; datlam10006@gmail.com (P.-D.L.); bonhyeon.gu@9bon.org (B.-H.G.); hoangkhanh9119@gmail.com (H.-K.L.)

**Keywords:** digital twin, smart city, CityGML, building information model (BIM), industry foundation classes (IFC), 3D visualization

## Abstract

The growing interest in building data management, especially the building information model (BIM), has significantly influenced urban management, materials supply chain analysis, documentation, and storage. However, the integration of BIM into 3D GIS tools is becoming more common, showing progress beyond the traditional problem. To address this, this study proposes data transformation methods involving mapping between three domains: industry foundation classes (IFC), city geometry markup language (CityGML), and web ontology framework (OWL)/resource description framework (RDF). Initially, IFC data are converted to CityGML format using the feature manipulation engine (FME) at CityGML standard’s levels of detail 4 (LOD4) to enhance BIM data interoperability. Subsequently, CityGML is converted to the OWL/RDF diagram format to validate the proposed BIM conversion process. To ensure integration between BIM and GIS, geometric data and information are visualized through Cesium Ion web services and Unreal Engine. Additionally, an RDF graph is applied to analyze the association between the semantic mapping of the CityGML standard, with Neo4j (a graph database management system) utilized for visualization. The study’s results demonstrate that the proposed data transformation methods significantly improve the interoperability and visualization of 3D city models, facilitating better urban management and planning.

## 1. Introduction

Within the context of the Fourth Industrial Revolution, the concept of a “Digital Twin” has significantly impacted urban planning by providing a virtual model that can simulate and analyze real-world behaviors. The technologies of the digital twin, operating at various levels of detail, enable the prediction of performance, process optimization, and cost reduction across sectors such as manufacturing, transportation, and energy [1,2,3]. Effective urban management and modeling are essential for fostering sustainable smart city development [4]. Therefore, it is critical to analyze the implications of urbanization and devise innovative solutions for the development of cities [5,6].

BIM provides detailed data about buildings that can be generated, stored, managed, exchanged, and shared in a flexible and reusable way [7]. These data help professionals from different industries collaborate more effectively, manage projects efficiently, and reduce costs. BIM also enables the creation of detailed 3D models of structures, serving as a unified platform for data and visualization. BIM enhances collaboration among all stakeholders, enables efficient coordination with integrated project delivery systems, ensures transparency, optimizes data integrity, and facilitates rapid communication and data exchange. Additionally, BIM integrates various principles through feasible communication, analyzes the construction feasibility of project systems, and assesses project costs and time [8].

In recent years, 3D models enhanced with semantic data have provided comprehensive virtual representations of physical assets or systems. These models integrate various data sources such as real-time sensor data, historical data, and simulation models. On the other hand, 3D GIS primarily focuses on the visualization and analysis of geospatial data in three dimensions, providing insights into real-world geographic features and phenomena. According to Barricelli et al. (2019) [9], digital twins emphasize the bi-directional relationship between the physical and virtual worlds, enabling continuous synchronization and a feedback loop for enhanced decision-making.

Digital twin data are commonly stored using CityGML, an XML-based international standard. CityGML is developed by the Open Geospatial Consortium (OGC) [10] as a 3D information exchange standard that facilitates the exchange of three-dimensional virtual urban models by defining core attributes and relationships; it is adaptable through the addition of new attributes, as required [11,12]. The 3D City Database was employed to configure a database for managing CityGML data, equipped with input/output tools and visualization capabilities. However, conventional web-based visualization services suffer from the limitation of requiring the server to hold all the data for the output [13,14,15].

The subsequent research has highlighted the need to consider specialized semantic linkage structures for inference when utilizing CityGML [16,17]. Such structures often take the form of graphs by employing knowledge representation techniques, such as production rules and logic-based representations. One notable solution within the realm of semantic linkage structures is the “Semantic Web”, a machine-readable web that interconnects vast and distributed data, enabling agents to read and infer from it. This indicates the suitability of the structure for inference [18]. In the context of digital twins, the prior research has explored the integration of the semantic web into IFC, a data management approach for building information. Through this integration, the transformation of BIM into an ontology using the semantic web has been proposed to enhance inference accuracy [19,20,21,22].

CityGML serves as an open data model for the depiction and exchange of 3D city models. In its current iteration, version 2.0, CityGML incorporates five distinct levels of detail (ranging from coarse LOD0 to highly detailed LOD4) to depict various features within city models, encompassing individual buildings to entire urban landscapes. CityGML 2.0 is widely embraced in both academic research and industrial applications due to its user-friendly nature and seamless integration [23,24]. However, with the expanding scope of its applications, an inherent limitation arises: the current classification system allocates all internal features to LOD4, necessitating intricate representations both externally and internally. This approach is insufficient for highly detailed and complex buildings, which require varying levels of precision for internal structures. In analyzing indoor environments, the impact on analysis results may be less influenced by external details. Thus, an appropriate definition of different complexity structures is necessary to apply, exchange, and outline data specifications between building components, in order to minimize loss of data.

In response to a growing population, numerous cities are implementing urbanization strategies and incorporating advanced technologies to streamline this process. Digital twins and BIM are notable concepts that involve the creation and analysis of 3D models. These models are utilized in various applications and scientific disciplines, including construction [25], policymaking [26], digital preservation [27], and healthcare [28]. The models are developed at varying levels of detail (LOD), thereby broadening their applicability across different fields.

In this study, the authors present data conversion methods within the context of Busan, South Korea, a smart city where residents’ quality of life is a paramount concern. This research aims to achieve three primary objectives. Firstly, the authors propose the conversion of IFC mapping classes to the CityGML standard at levels of detail 4. Secondly, an OWL/RDF ontology is introduced to transform the CityGML structure into RDF format, enabling the creation of a semantic representation for the web. This transformation facilitates more efficient semantic-web-based inference, thereby enhancing the depth and accuracy of the proposed methods. Lastly, this study visualizes Internet of Things (IoT) sensor data by converting comma separated value (CSV) data to keyhole markup language (KML) format. Subsequently, platforms such as Cesium Ion and Unreal Engine are introduced for realistic data visualization, enabling the visualization of geometric values and information within 3D data models. Additionally, the analysis, visualization, and evaluation of OWL/RDF ontologies are conducted through the Neo4j database.

## 2. Materials

### 2.1. Semantic 3D City Data Model

The design of a three-dimensional data model for building-type structures using IFC, land extensible markup language (LandXML), 3D feature geographic markup language (3DF-GML), graphics library transmission format (glTF), keyhole markup language (KML), IndoorGML, CityGML, and other representative international standards [29,30,31] was explored.

Kim et al. (2018) conducted a study to compare and analyze a group of three-dimensional data models that are most commonly used in academia and industry, and the criteria used in this research are classified as geometry, topology, texture, level of detail, semantic, attribute, and geo-reference [32]. The comparison and analysis criteria include geometry (3D geometric information), topology (topological information), texturing (mapping real-world images to spatial objects), level of detail (LOD) (the detail level of 3D objects), semantic information (conceptual data model), and data attributes and georeferencing (Geo-ref.). Based on these criteria, this study conducted a comparison and review of the international standards for 3D data models by adding criteria such as history management (versioning), sensor information representation (sensor), and indoor space representation (indoor), which are important for data models related to building-type structures.

As a result of comparing and analyzing the above items among three-dimensional data models, CityGML was judged to be the most suitable data model as a reference framework for data model development because it supports all of them and can flexibly extend the model, as shown in Table 1. Furthermore, CityGML comprehensively covers various objects that make up a city, such as bridges, tunnels, and roads, in addition to the buildings covered in this study, and enables efficient modeling of three-dimensional objects at macro and micro scales. In addition, as an official standard of the OGC, which deals with international standards for spatial information, it can be highly reliable, and for this reason, it is already used as a reference framework for developing 3D data models in many countries [33,34,35].

### 2.2. Applications Domain Extension for Smart City

Interoperability between CityGML implementations for public infrastructure and geographic information has been greatly enhanced by the introduction of the Infrastructure for Spatial Information in Europe (INSPIRE) application domain extension (ADE) [36]. The INSPIRE ADE was developed within the building data specification of the INSPIRE standard, focusing on 3D buildings and related attributes such as building addresses, characteristics, and data quality. Batty et al. (2000) [37] and Biljecki et al. (2018) [38] presented a conceptual framework for 3D city models, focusing on visualization and spatial planning. Their taxonomy categorizes 3D city models into 12 distinct industries covering areas such as e-commerce, telecommunications, education and learning, real estate analysis, city portals, marketing, economic development, facilities and utility management, tourism, entertainment, urban planning, environment, emergency services, and architecture.

InfraADE [39], developed by Shen et al. in 2020, is a framework that represents a novel combination of LandInfra and CityGML concepts. It is a relatively recent addition to the OGC standards and aims to harmonize land and infrastructure functions while incorporating key principles of BIM and geographic information systems (GIS). The scope of LandInfra, which includes various elements such as buildings, roads, railroads, and terrain, is very similar to CityGML [40,41]. Zadeh et al. [42] introduced a conceptual approach for developing hybrid information infrastructure by integrating building design data, in the form of *ifc*XML and 3D neighborhood models, in the form of CityGML.

In 2021, Braun et al. [43] utilized 3D CityGML to model food waste and wastewater patterns in the city of Montreal. Employing the 3D CityGML framework, they conducted simulations to discern trends in waste generation and dispersion throughout the urban landscape. Their study proposed strategies to pinpoint and alleviate areas with heightened waste production. Concurrently, Biljecki et al. [44] investigated the disparities in data representation between IFC and CityGML, presenting a method for converting IFC data into CityGML format. This conversion process effectively showcased how CityGML can be enriched with information derived from IFC-based 3D city models. The findings indicated that CityGML provides a more comprehensive representation of 3D city models, enhancing their utility in various applications within the realm of urban planning and management.

The GRextADE system, developed by Theodoros et al. [45] in 2022, was designed to address the specific requirements of 3D modeling within the urban landscape of Greece. It aims to tackle challenges arising from an incomplete cadastre and the absence of infrastructure for effective data management, particularly in facilitating the 3D visualization of urban data. Chadzynski et al. [46] introduces a system architecture utilizing CityGML-based conceptual schema and intelligent autonomous agents to build scalable information systems for large city modeling, enabling dynamic geospatial knowledge graphs and addressing pitfalls of Web 2.0 applications while integrating artificial and human intelligence.

### 2.3. Integration between IFC and CityGML Model

The approach involves unidirectional transformation, converting IFC building models into CityGML models. Dongkers [47] devised a methodology for converting LOD4 building models into the CityGML format. This process involved extracting and mapping the IFC semantics to CityGML semantics, followed by geometric generalization, which extracted the exterior shell using Boolean and morphological operations. Subsequently, semantic and geometric refinements were applied to optimize the model for analysis. The prototype implementation demonstrated the efficacy of the methodology while also highlighting limitations arising from missing information in IFC’s semantics.

An approach was devised to integrate an IFC model with a CityGML model through the semantic and geometric generalization of the IFC models, implemented as a prototype within the IFCExplorer software by Geiger et al. [48]. The initial step involves generating an intermediate data model using the ExtrusionBaseModel, focusing solely on pertinent building elements. Each selected building element is represented by its footprint to establish a standardized geometric foundation, with extrusion containers subsequently calculated based on these footprints. Additionally, extrusion containers for building stories are generated. This ExtrusionBaseModel, inclusive of the extrusion containers, serves as the foundation for all subsequent transformations. Testing has been conducted on simple house models, with plans to extend testing to more complex buildings in the future.

In 2016, Deng et al. [49] introduced mapping rules between IFC and CityGML utilizing an instance-based method. They also crafted a reference ontology and a CityGML application domain extension (ADE) for schema mediation. Their method underwent testing, which demonstrated the accurate geometric transformation of building components and preservation of semantic information from IFC to CityGML. However, this study’s scope was restricted to geometry, as only three types of geometric construction in IFC were taken into account for the transformation. In the same year, Karan et al. [50] improved the data exchange and integration between BIM and GIS, transitioning from a syntactic to a semantic level by incorporating data semantics. They developed a new ontology based on the EXPRESS schema at the application level, known as BIM ontology. This ontology facilitates the seamless integration of building- and construction-related data, encompassing all IFC classes with their respective attributes. However, disparities in levels of detail between BIM and GIS ontologies can impede data and information sharing quality. Consequently, many elements of IFC buildings cannot be semantically transferred into the GIS model.

In this research, the developed transformation system utilizes FME version 2023.1 by Safe Software. FME is well-known for its proficiency in spatial extract, transform, and load (spatial ETL) processes. FME efficiently translates spatial data between various digital formats, enabling extraction from the source data, necessary transformations for usability, and loading into destination views or datasets. It supports diverse file formats and databases, particularly focusing on 3D models and geographical information systems, including CityGML, Autodesk 3DS, Collada, and ESRI shapefiles, among others. FME’s flexibility extends to bridging the gap between different file formats, facilitating tasks such as converting lidar images into simplified 3D models. Furthermore, the FME server offers additional functionalities, such as application programming interfaces (APIs) and web-based management, allowing developers to create real-time data conversion applications using Python. The introduction of an FME Server playground enables developers to explore various possibilities for utilizing FME server capabilities.

This study presents a robust ETL workflow designed for seamlessly integrating IFC, CityGML, and FME. The workflow incorporates well-defined transformations between IFC and CityGML models at LOD4, as well as between IFC and FME. By leveraging these ETL workflows, the process of integrating IFC with CityGML/FME is streamlined, offering users the flexibility to develop their data mappings. This approach ensures versatility in usage without necessitating specialized software expertise.

## 3. Methods

This study introduces a process for converting the original BIM data to CityGML and describes the parameterization of the building as a 3D city model in CityGML in Section 3.1. The proposed conversion process ensures the generation of 3D models that comply with the five LOD levels of detail specifications defined in CityGML (Biljecki et al., 2016) [51]. This provides for the generation of different representations for each building, distinguished by their geometric complexity, as well as the creation of models that span multiple geometric references. For example, as discussed in Biljecki et al. [52], it provides LOD2 models with walls placed exactly at their actual location and alternate versions of walls projecting from the roof edge. This includes the creation of models with different levels of semantic structure, which is exemplified in both LOD3 models with and without thematically rich surfaces. It also includes models that represent the distinction between geometric types, including both boundary and shape representations. The transformation tool can generate corresponding room geometries across multiple LODs in Section 3.2. In Section 3.3, the authors use Neo4j to validate the transformation process from CityGML to RDF/OWL graphs.

The methodological framework consists of three detailed components: data transformation, building sensor data mapping rules, and data visualization.

- Data Transformation: In this component, data are converted from IFC and CityGML schema to generate semantic mapping candidates. These candidates are then stored in GML format.
- Building Data Mapping Rules: The semantic mapping rules created in the first component are integrated with an RDF graph ontology, which allows the association of multiple open structures.
- Data Visualization: The original IFC data are transformed into a CityGML model based on the mapping rule constraints and visualized through web services. To visualize the CityGML model on the Cesium Ion and Unreal Engine platform, it is necessary to convert CityGML into 3D tiles.

**Figure 1:**
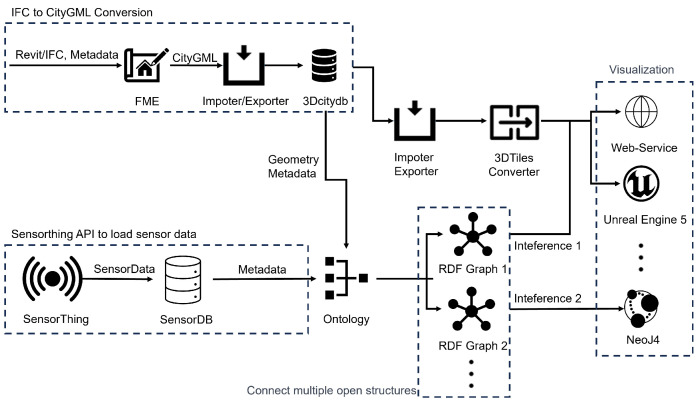
The detailed workflow of the proposed methodology for data visualization in city modeling.

The main steps of this methodology are illustrated in Figure 1.

### 3.1. An Approach to the Proposed Visualization Process

In order to simplify (serialize) the generation of multi-level of detail (LOD) data, it is necessary to analyze the structure of 3D BIM data and sensor data. The proposed city model and sensor data visualization process are shown in Figure 1, and the details are as follows. (1) First, the 3D building data are converted into CityGML standard format and GML data by a standard conversion table between classes using the FME tool and integrated standardized data. The integrated standardized data are securely stored in the 3D City Database, while the sensor data are kept separate to ensure the integrity of the geographic attribute processing [53,54]. (2) To facilitate the correlation between the geographic information of the sensor data and the 3D building data, an ontology of the data is built so that they can be extracted and represented as RDF graphs. Open-source platforms such as TerriaJs, Cesium, Neo4j, or Unreal Engine can be used to perform contextual visualization. In particular, Neo4j can efficiently analyze data in the form of RDF graphs, enabling complex semantic queries and greatly enhancing the exploration of data relationships. The detailed visualization process is shown in Figure 1.

### 3.2. Transforming 3D City Models into CityGML 2.0 Objects

**Figure 2:**
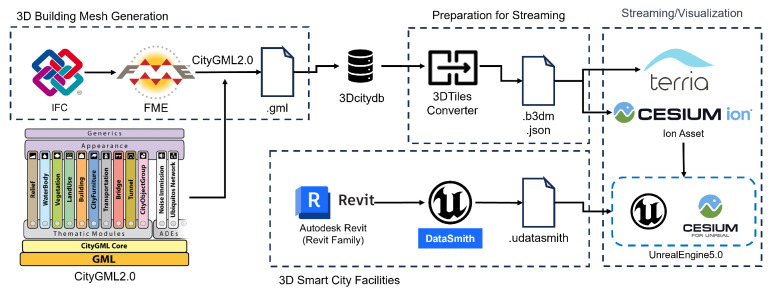
Workflow of the 3D mesh generation process for visualization scenarios.

Figure 2 outlines the data conversion process for the web visualization of 3D building model data, leveraging input from Revit files covering building entities and smart village facilities.

#### 3.2.1. Semantic Mapping from IFC to CityGML at Level of Detail 4

**Figure 3:**
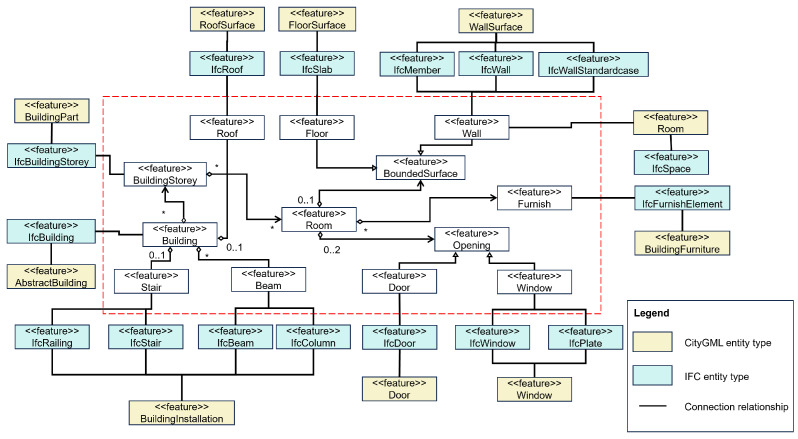
The semantic mapping rule between IFC and CityGML entities. Note: “*” UML notation used to representation the cardinal relationship among CityGML classes that shows the number of occurrence or possibilities and an intermediately model is shown inside the red box.

A formal semantic mapping procedure is introduced to facilitate the correspondence between individual instances belonging to the industry foundation classes (IFCs) and their counterparts within the CityGML class. This mapping process considers various aspects including entity interrelationships and attributes. This procedural framework is depicted in Figure 3, where both IFC and CityGML entities are seamlessly integrated into a unified modeling representation, often realized through the application of unified modeling language (UML) techniques. The demarcated region outlined by the dashed red lines encompasses a pivotal component known as the bridge model. This model functions as an intermediary, establishing a cohesive link between entities originating from the IFC and CityGML realms. Importantly, this bridge model ensures a comprehensive transformation of data between these entities by utilizing intermediate objects, thus facilitating accurate and consistent information exchange.

#### 3.2.2. The Intermediate Model-Based Data Transformation

The first step in the data conversion process is the conversion of the building data format based on the IFC 2 × 3 coordination view 2.0 standard, as mentioned before in the 3D building mesh generation process in Figure 2. In other words, the IFC to CityGML conversion step is performed by the feature manipulation engine (FME), an extract, transform, and load (ETL) mechanism, and an integration process involving the standardized CityGML schema is performed [10]. Furthermore, the assimilation of building information and the related location coordinates is extended to include the 3DTile format for the visualization of the city model. The visualization of the city model is implemented on different platforms such as TerriaJS and Cesium Ion.

In the second phase, the conversion switches from Revit to Datasmith for realistic graphical depiction. This is represented by a collection of tools and plugins that can seamlessly import complex assets and pre-built scenes from various design applications into a 3D graphics engine such as Unreal Engine. This transition covers a wide range of industry-standard design applications, enabling integration within environments like Unreal Engine.

The detailed data conversion process using FME Workbench is as follows.

In processing IFC data, it is essential to establish a hierarchical relationship between IFC features and their respective parents, including the removal of intermediate features such as openings [55,56]. It is essential to establish a hierarchical relationship between IFC features and their respective parent features. This requires two IFC data parsing steps.

The initial IFC parsing step is as follows: A detailed lookup table is created to extract all IFC features as a first IFC reader. The detailed lookup table contains a secondary table that catalogs the parent feature types, along with the associations between feature IDs and parent IDs. Notably, this initial parsing step excludes geometry information for fast execution, and feature data for fast execution. (Note that the data processing platform, FME, orders these parsing steps to ensure completion of the first step and to ensure complete lookup tables before starting the second step).

**Figure 4:**
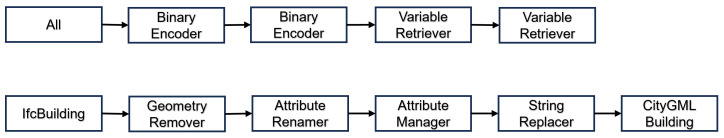
Parent/child lookup features (**above**) and conversion from IfcBuilding to CityGML Building (**below**).

The second IFC parsing step focuses on data transformation. This step includes the process of simplifying building features by removing associated geometry through the placement of the “GeometryRemover” transformer [57]. Additionally, the “AttributeRenamer” transformer is used to set the ifc_unique_id attribute to match gml_id. Figure 4 shows this process.

#### 3.2.3. IFC-CityGML Transformation for Representative Classes

The data conversion process involves basic IFC-CityGML correspondence classes such as IfcBuilding–Building, IfcDoor–Door, IfcWindow–Window, etc.

- First, obtain the Ifc class data (geometric and properties) from the Ifc class, and then convert the complex solid data to multi-surface using ConvertGeometry.
- Next, set the gml_id when changing the ifc_unique_id property and add a classification keyword such as gml ID (gml:id= “door_2GpVABPS5EtPLZHNRXT_286216”).
- Create a CityGML master link for Building using the custom variable GetGrandParentID generated by the detailed lookup table.
- Build the data into the final CityGML by setting the LOD and feature roles for the CityGML.

**Figure 5:**
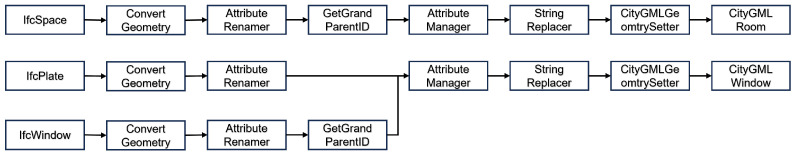
A simple conversion, converting IfcSpace to CityGML Room (**above**) and IfcPlate–IfcWindow to CityGML Window (**below**).

The above data conversion process is shown in Figure 5.

**Figure 6:**
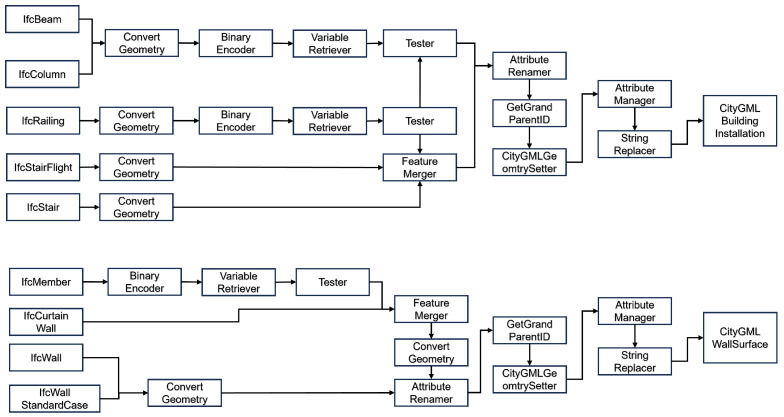
Complex conversion of CityGML Building Installation (**above**) and Wall Surface (**below**).

A more complex scenario occurs when the “BuildingInstallation” and “WallSurface” entities are composite structures composed of different IFC feature types, and the conversion process is as shown in Figure 6. In particular, certain IFC features show the possibility of dual membership in the construction of “BuildingInstallation” and “WallSurface”. In the construct of “BuildingInstallation” and “WallSurface”, for example, the combination of “IfcMembers”, “StairFlights”, and Railings creates a Stair entity, which is then converted to a “BuildingInstallation”. This complex also includes “ifcColumns” and “ifcBeams”. Additional IFC Members are combined to form “ifcCurtainWalls”, which are then represented by a “WallSurface”.

### 3.3. CityGML to RDF Graph Conversion

To validate the CityGML conversion model within the proposed IFC model, it is essential to conduct the RDF graph conversion process for CityGML data. This conversion process ensures accuracy verification and alignment with the linked data, thereby guaranteeing semantic compatibility. This facilitates integration between different source data and validation against ontologies and domain-specific rules. Ultimately, the effectiveness of the proposed methodology can be validated by improving the accuracy, reliability, and accessibility of the transformed CityGML data within the linked data through RDF-based representation.

#### 3.3.1. CityGML Tree Structure

In order to represent a comprehensive CityGML city model, the model should begin with the tag “CityModel”, rather than a partial model, and the various sub-models should be described. Within the CityGML framework, a terminal segment that has no further children should own a value, unless the information is unknown or undocumented. For the scope of this investigation, this research assumed that there are no unknown or unmentioned data elements within CityGML to minimize the need for exceptional handling.

**Figure 7:**
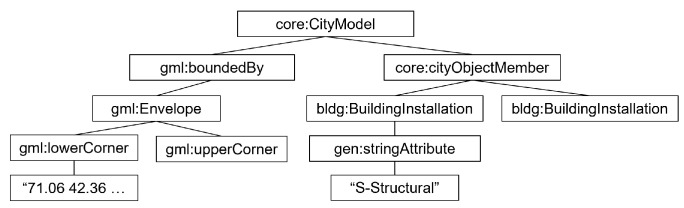
The hierarchical CityGML tree structure of building-type community center sample.

The structural features of CityGML are tree-like, including the presence of root and leaf nodes, and nodes other than root have an exclusively singular parent. Figure 7 demonstrates nodes in the CityGML structure tree.

#### 3.3.2. Organizing Link Data: Creating a Tree Structure

**Figure 8:**
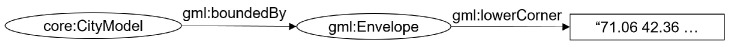
Example of CityGML Building envelope and literal.

In the first rule, instead of describing fragmented models within CityGML, the modeling approach starts with an encapsulated element, “CityModel”. This facilitates the description of multiple complex models that collectively comprise a comprehensive and standardized city representation within the CityGML framework. In addition, the hierarchical structure within CityGML ensures the logical integrity of the model’s hierarchical organization, with leaf intervals being accompanied by correct attribute values when additional child elements are no longer integrated. This can be seen in Figure 8.

**Figure 9:**
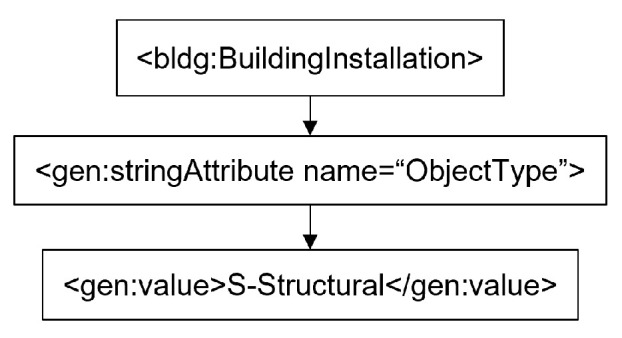
Illustration of Attribute Structure in CityGML: A Generic Example.

The second convention is the handling of attribute tags within the CityGML generic class, which are used to organize detailed representations and metadata. These elements allow for the creation and utilization of variables by composing an unlimited number of pairs of names and values. Similar to the creation and application of variables, attribute tags provide the flexibility to unambiguously express pairs of names and values. These tags also consistently encompass the “name” attribute and its “value” tag as obligatory children to facilitate association. Figure 9 shows an example of an “Attribute” relationship.

**Figure 10:**
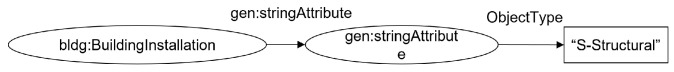
The candidate attributes rules of the CityGML structure sample.

In the context of representing the above CityGML tree structure in RDF, there are two rules: (1) to use the attribute tag as a node with “name” as the predicate and “value” as the object and (2) to represent “name” and “value” as literals and concatenate them into a BNode, as shown in Figure 10.

**Figure 11:**
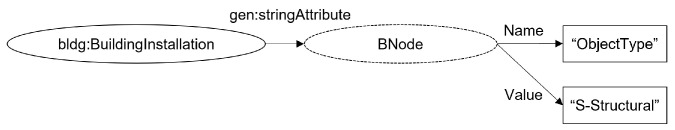
The candidate attributes rules of the RDF structure sample.

Figure 11 represents an advantage of the simplicity of the RDF structure. However, the variable nature of the predicate variations based on “name” within this rule may cause normalization problems, which has the potential to reduce data integration and query efficiency. In contrast, Figure 11 utilizes BNodes to clearly represent the relationship between “name” and “value”, and in the proposed process, attributes are transformed by BNodes.

**Figure 12:**
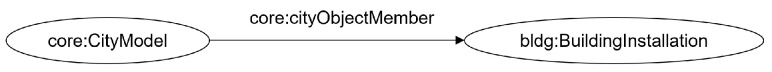
Example of predicate registration in CityGML-IFC core: cityObjectMember is a predicate.

The third rule is to always translate CityGML elements whose values are not explicitly provided into predicates in RDF. For example, “core:cityObjectMember” refers to a relationship that considers the element “cityObject” within the parent element to be a child element. During the transformation process, this relational information is utilized as a predicate to define the triple element in RDF. This third rule is illustrated in Figure 12.

**Figure 13:**
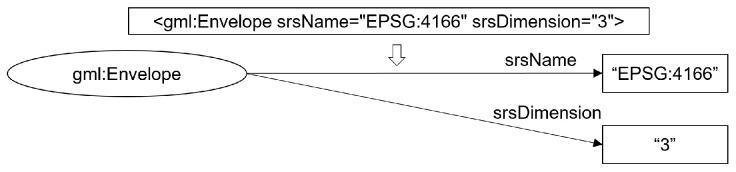
Example of attribute registration within a CityGML tag: an attribute example with gml:Envelope.

The fourth rule pertains to the handling of internal attributes within each element tag of the CityGML. In certain instances, these attributes may encompass information unrelated to the RDF, such as the IDs assigned by the CityGML generator. However, if these attributes hold pertinent information to be included in the RDF (e.g., meaningful metadata), they should also be transformed into literals. This transformation was executed using the definitions provided by the user. An example is shown in Figure 13.

### 3.4. CityGML 2.0 to 3D Tiles Approach

The conversion process begins with the input BIM data in IFC format, serving as the initial step. These data undergo a conversion process and are stored in CityGML format, representing LOD4. Following this transformation, the CityGML data are further converted into 3D Tiles format, ready for streaming purposes. This streamlined process ensures the seamless transition from BIM to a dynamic and interoperable 3D Tiles format, facilitating efficient data utilization and visualization.

**Figure 14:**
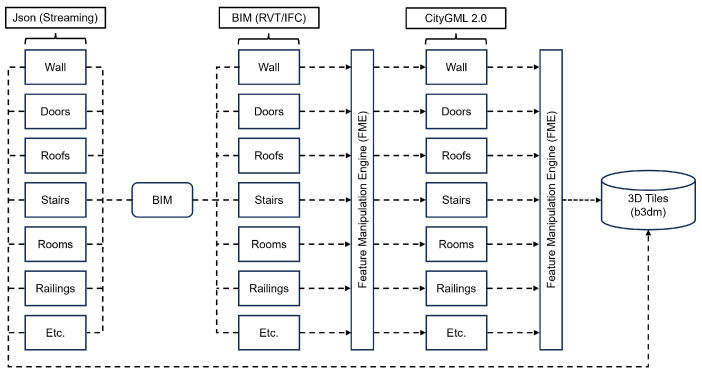
The workflow involves converting CityGML data into the Cesium 3D Tiles format.

In the initial step, all components within the IFC model are converted and saved as a CityGML file, as described in Section 3.1. In the next step, this CityGML file is converted into a single composite or multiple b3dm files. These files are organized in a hierarchy of batch tables and are accompanied by a tileset.json file that collectively defines the 3D Tile format. Figure 14 is a visual representation of a comprehensive workflow that illustrates this process.

Cesium 3D Tiles is a geospatial data format and rendering engine that plays a pivotal role in the visualization and streaming of 3D geospatial content over the web. It provides an efficient and scalable way to deliver large-scale 3D geographic information, enabling the interactive exploration of complex 3D environments within web-based applications and virtual globes. Cesium 3D Tiles excels at streaming and rendering complex 3D models in real-time and with efficiency. However, they may not be optimized for storage and can cause problems when dealing with very large models. The authors addressed such a scenario by implementing a process for converting from CityGML to Cesium 3D Tiles.

**Figure 15:**
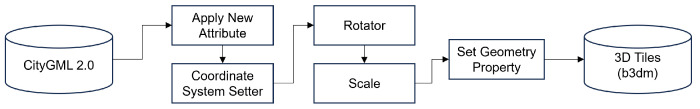
The implementation process from CityGML to Cesium 3D Tiles.

The conversion process involves a few key steps. First, on the New Properties tab, update the information that is important for building identification, including attributes such as name, type, and related parameters. Next, utilize the CoordinateSystem tab to utilize EPSG codes and corresponding coordinates to ensure accurate geospatial placement on the Cesium world map. Since the model can be quite large and needs to be oriented when uploaded to Cesium Ion, the Scaler and Rotator tabs are used to manage scaling and rotation along the x, y, and z dimensions. Finally, the resulting data are saved as a bd3m file. To facilitate searching and querying the data uploaded to Cesium Ion, the authors utilize JSON files as a standard tool for accessing and interacting with the storage. This process is illustrated in Figure 15.

### 3.5. Integrate Sensor Data into CityGML Model and KML Visualization

KML, a markup language built upon the XML standard, employs a structured markup format comprising nested elements and attributes to facilitate geotagging. Widely utilized in software applications like Google Earth, Google Maps, and Cesium Ion, KML files adhere to the KML language specifications. Notably, KML also enables the visualization of sensor data as time series, extending its utility beyond basic geospatial representation. Its support for interactive querying enhances the user experience by simplifying the retrieval of sensor data quality information. Additionally, KML’s capability to incorporate gradually varying colors allows for the intuitive highlighting of sensor data quality levels. This versatile nature of KML empowers users to effectively visualize, analyze, and interact with sensor data, making it a valuable tool in various geospatial applications and platforms.

**Figure 16:**
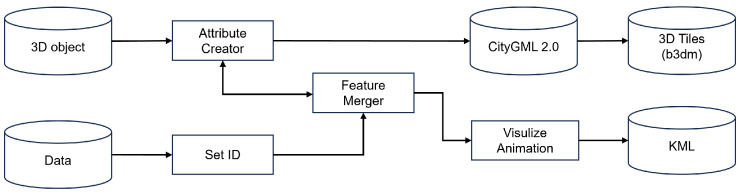
Visualizing 3D Objects with Metadata Animation Using KML in the Cesium Web Service.

Proper sensor data processing is achieved through the FME workbench, illustrated in Figure 16. Initially, a 3D position sensor model is provided with only the sensor ID and name attributes. However, this model lacks the necessary metadata integration. To address this, the AttributeCreator conversion tab is incorporated to generate the required metadata based on the second input. The second input encompasses sensor data information, including metadata such as sensor ID, measurement interval (start–end), location, temperature, humidity, and fine dust. The FeatureMerger transformer tab serves the crucial function of linking metadata to the 3D model via the common attribute of the sensor ID. Leveraging this attribute, the metadata are seamlessly integrated into the 3D model, which is then stored in CityGML 2.0 format and converted to 3D tiles for visualization on a web platform. To enhance data visualization, animated representations are generated and stored for online visualization purposes on a web platform. The animation’s color scheme dynamically adjusts based on internal sensor data, such as temperature, humidity, or other measurement parameters. Finally, the data are saved in KML format for optimized online visualization. This approach ensures efficient and accurate processing of sensor data, facilitating comprehensive visualization and analysis on web platforms.

## 4. Results

In the context of Busan Smart City, this study identifies BIM as a pivotal and indispensable technological avenue for enhancing the construction and management of urban environments. BIM offers substantial prospects for fostering sustainability, efficiency, and overall enhancement of quality of life. BIM plays a pivotal role in ensuring that the infrastructure of a smart city is tailored to address the specific needs of the community while considering important factors such as energy conservation, resource optimization, and resilience to climate change impacts. BIM also facilitates seamless collaboration between the various stakeholders involved in a construction project, mitigating the risk of errors and costly rework and supporting informed decision-making.

### 4.1. BIM(IFC) to CityGML: 3D Mesh Generation

In this research, the authors implemented the data integration conversion method from IFC to LOD4’s CityGML. Specifically, the process is performed on a dataset consisting of 56 individual building models in the IFC format.

**Figure 17:**
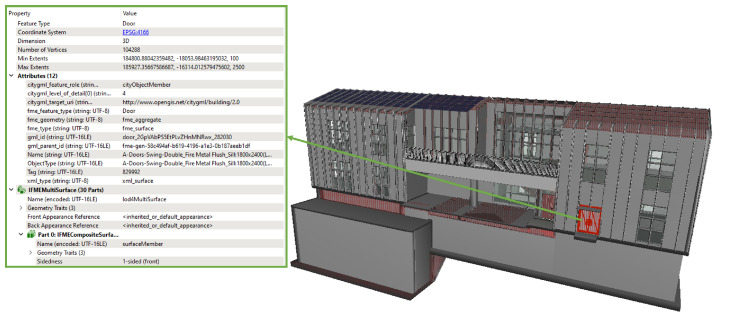
3D geometry and properties results of the CityGML model via FME Inspector.

The transformation of 3D BIM data into CityGML-compliant models was successfully achieved through a detailed process involving semantic mapping and the utilization of FME tools for data translation. This approach ensured the accurate and comprehensive representation of urban features within the models. To verify the effectiveness and accuracy of the proposed conversion method, the authors aimed to ensure a smooth transition of data from IFC to CityGML in LOD4. Figure 17 illustrates the visual output, depicting the generation of building objects conforming to the specifications outlined in the CityGML LOD4 schema. In this figure, the right side highlights a selected 3D building element in red, while the left side displays detailed properties such as gml_id, Levels of Detail, feature role, and name associated with the highlighted element. Table 2 provides a comprehensive overview of the sub-nodes comprising the generated CityGML object, organized based on their designated functions. The XML description included in Table 2 outlines the door component, which is referenced by the door using Xlinks to elucidate the topological relationship between the door and the building. The coordinates indicate the geometric parent of the building, and the relationship properties detailed in the metadata information offer further insights into the door component.

**Figure 18:**
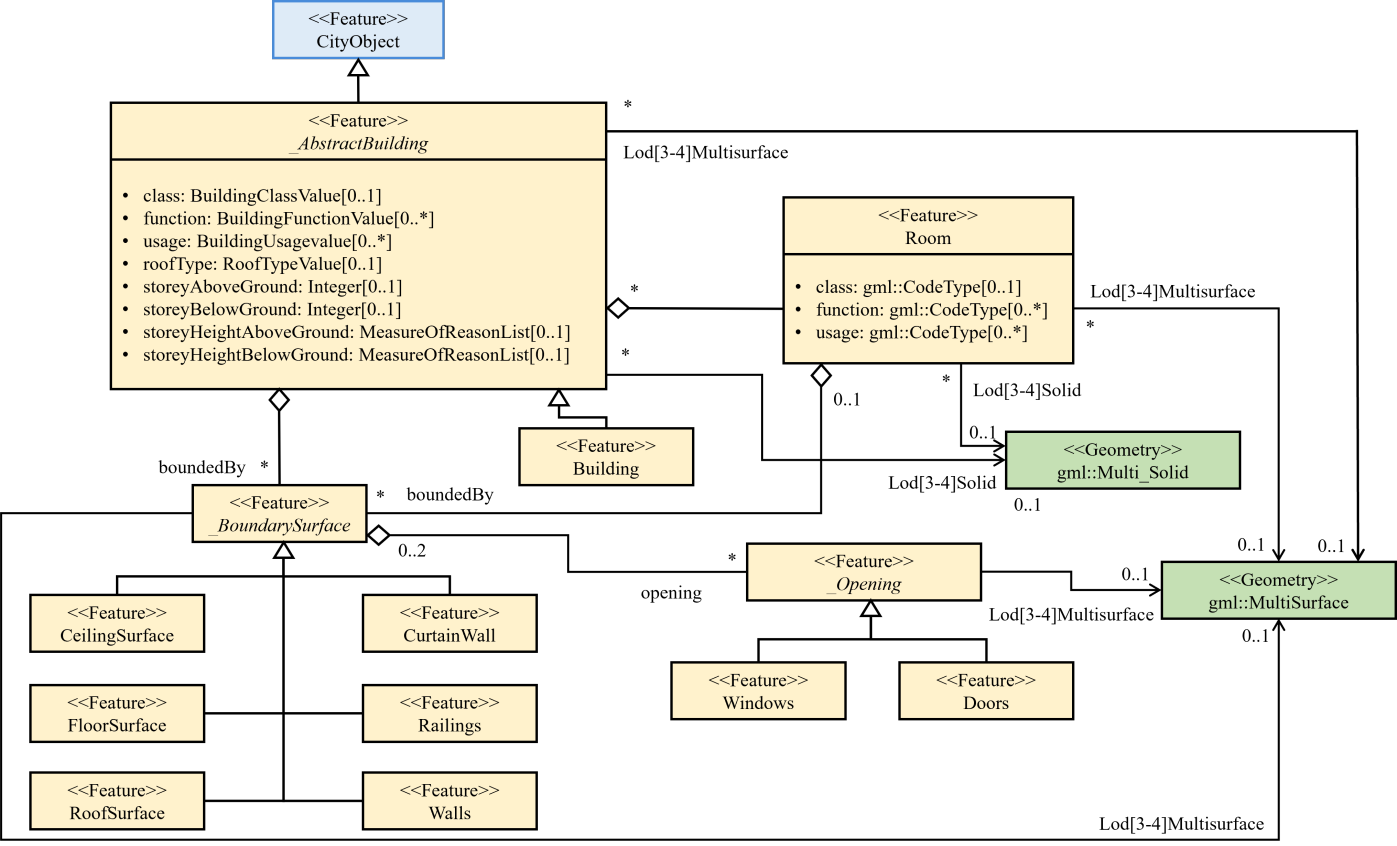
3D geometry and properties results of the CityGML model via FME inspector.

The UML diagram presented in Figure 18 illustrates the various methods for defining the geometry of a building, showcasing the model’s refinement from LOD3 to LOD4. Consequently, not all components of a building model are equally represented in each LOD, and certain aggregation levels are restricted to specific LODs. In CityGML, all object classes are linked to the LODs based on the minimum acquisition criteria required for each level. At the core of this structure is an abstract class named “_AbstractBuilding”, which serves as a subclass of “_cityObject” within the core module of CityGML. The feature room is depicted by a geometric primitive called Solid, which is enclosed by its boundary surfaces. The semantic class “_BoundarySurface”, as defined by CityGML 2.0, is utilized for boundary surfaces, including “CeilingSurface”, “Floors”, “Railings”, “Roofs”, and “Walls”.

BIM models are meticulously crafted, containing comprehensive information on every building component encapsulated within its modeled elements. Despite the intricacy of the models, the transformation workflows adeptly manage them, ensuring no loss of information or incorrect geometry. Nevertheless, during the transformation process, the file sizes expand, leading to variations in sizes between BIM models, IFC, and CityGML models. Notably, the most significant increase in file size occurs when generating LOD4 CityGML files, as illustrated in Table 3. Consequently, processing these complex building models and rendering their visualizations necessitate high-performance computers.

### 4.2. CityGML to RDF Graph

**Figure 19:**
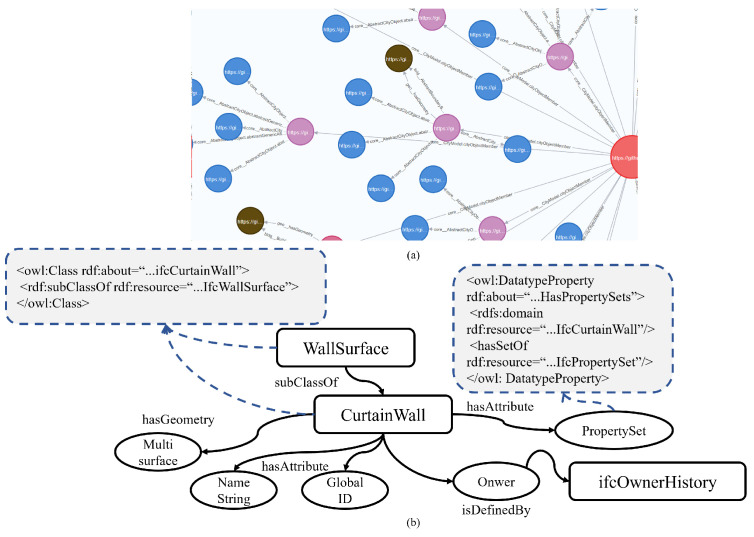
Graph representation for CityGML: (**a**) RDF Neo4j sample and (**b**) RDF graph and OWL classes of IfcCurtainWall entity.

The RDF/OWL-based semantic graph enabled advanced querying and inference, significantly enhancing the ability to analyze complex relationships within the urban environment. This capability is crucial for decision-makers, who need to assess the impact of various factors on urban planning and management. The formalized definitions for the RDF transformation of the community center sample’s CityGML schema are depicted in Figure 19a as a Neo4j visualization, while Figure 19b shows the RDF graph of the “IfcCurtainWall” entity of this building and its correspondence between RDF and OWL classes. In this experiment, the authors utilized the Neo4j graph database with the neosemantics plugin to visualize the RDF of CityGML. The graph centers on the basic building node “bldg_Building”, with element nodes adhering to the original CityGML structure as previously suggested. Predefined paths, constructed based on the predicates and logical relationships between elements, are essential for navigating from one element to another within the graph.

### 4.3. 3D Visualization View

**Figure 20:**
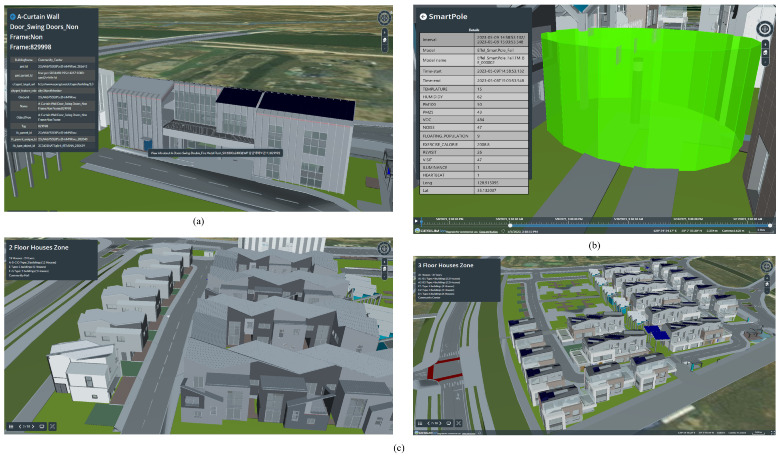
(**a**) Highlighted individual building elements in the Converted 3D Tiles of Cesium Ion and queried properties of highlighted building elements, (**b**) visualization of time series data, (**c**) visualizations of 2-floor models (**left**) and 3-floor models (**right**).

By integrating sensor data into the CityGML model and utilizing Cesium Ion and Cesium for Unreal Engine, the framework facilitated the real-time visualization of dynamic urban phenomena, including monitoring environmental parameters such as temperature, humidity, and air quality and visualizing their impacts on urban infrastructure. To verify and validate the accuracy of the proposed conversion method, the authors first visualized the model using Cesium Ion, a web visualization service that allows users to import and query 3D CityGML models. Figure 20a summarizes the results in the 3D Tile format for each model, visualized as CityGML attributes. The visualization of time series data with Cesium Ion highlighted certain constraints related to the types of simulations the Digital Twin platform can accommodate and limitations on the available data. For this experiment, the authors focused on sensor data recording ambient temperature over a three-day period, as shown in Figure 20b. Additionally, Figure 20c provides a visual representation of the area, distinguishing between sections with two-story houses (classified as ABCD, E, F–G) and three-story houses (classified as A–B, C, D).

**Figure 21:**
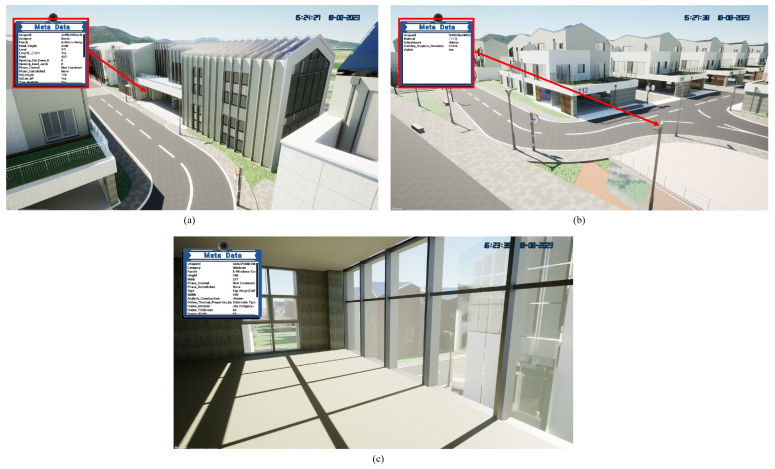
(**a**) Building element and (**b**) road furniture model and (**c**) the inside building in Unreal Engine 5.0.

Cesium Ion is a web service primarily designed for visualization purposes, excelling at providing a high-level overview of geographic data and structures. However, it has limitations when it comes to zooming in on building elements, often resulting in broken layers within the building model and restricted movement when navigating inside the structure. This limitation means that while Cesium Ion is effective for general visualization, it lacks the fine-grained detail necessary for seamless exploration and interaction within complex building environments. Unreal Engine 5.0 offers a robust solution to these limitations by providing advanced capabilities for rendering detailed building interiors and surroundings. Unlike Cesium Ion’s restricted functionality, Unreal Engine 5.0 empowers users with immersive interior views of buildings, enriched with comprehensive information about the structure and its environment. As depicted in Figure 21, this advanced engine enables users to explore intricate interior spaces in detail and interact seamlessly with the surroundings, offering a vastly enhanced experience compared to the limitations posed by Cesium Ion.

## 5. Conclusions

This study presents a semantic mapping framework for integrating IFC into the CityGML domain. The extract, transform, and load (ETL) software FME was employed to generate a transformation schema, thereby achieving comprehensive and enhanced semantic interaction capabilities.

The goals of this research can be summarized in three points:

- Firstly, the authors aim to address data exchange and integration between BIM and GIS from syntax to semantic levels by providing semantic data representations. The proper conversion of geometry and data information enables tackling the issue of IFC/CityGML information interoperability.
- Secondly, the research focuses on the application of RDF/OWL ontology, which establishes relationships among CityGML entities for RDF graph visualization.
- Thirdly, the research endeavors to visualize models for web and Unreal Engine applications.

A significant aspect contributing to the advancement of the research pertains to the adoption of Version 2 of the CityGML standard. Its potential lies in its harmonization with the INSPIRE standard building elements and its integration with Level of Detail 3 and 4 of the BIM models. Therefore, potential future advancements of the platform could entail the incorporation of this updated standard version, necessitating a review of the database structure while ensuring the retention of the existing maintenance data.

Another important aspect is that OWL offers robust rules for RDF to facilitate the generation and inference of new RDF data, a process commonly referred to as ontology deployment. Nevertheless, accomplishing this task necessitates not only establishing logical definitions for CityGML classes such as Building, Core and Common but also defining all classes and their interrelationships, demanding a thorough understanding of the domain.

The limitation of the present study consists of the methods proposed, tailored to the second version of the CityGML domain, are rendered entirely impractical for the third version. Furthermore, persisting issues include the occurrence of model noise, prolonged data transmission times on the web, and challenges in accurately positioning the model on the terrain surface.

## Figures and Tables

**Table 1:** The comparison between 3D data models.

Division	IFC	LandXML	3DF-GML	glTF	IndoorGML	CityGML2.0
Geometry	++	+	+	+	++	++
Topology	+	+	0	0	++	+
Texture	−	+	+	+	0	+
Versioning	+	0	0	−	−	+
Sensor	+	−	−	−	−	+
Indoor	0	−	−	0	++	++
LoD	++	0	+	+	+	++
Semantic	++	0	++	−	++	++
Attribute	+	+	+	−	0	++
Geo-ref	++	0	0	0	++	++

−: Not supported, 0: Basic. +: Supported, ++: Extended support.

**Table 2:** CityGML documentation of the example community building.

**Coordinate**
<gml:boundedBy>
<gml:Envelope srsName=“EPSG:4166” srsDimension=“3”>
<gml:lowerCorner>−29022.891454012053 181057.14042205806 −9774</gml:lowerCorner>
<gml:upperCorner>13290.79220452929 209229.45201190276 11000</gml:upperCorner>
</gml:Envelope>
</gml:boundedBy>
**Metadata Information**
<core:cityObjectMember>
<bldg:Door gml:id=“door_2GpVAbPS5EtPLvZHnMNRxT_286216”>
<gen:stringAttribute name=“GlobalId”>
<gen:value>2GpVAbPS5EtPLvZHnMNRxT</gen:value>
</gen:stringAttribute>
…
<bldg:lod4MultiSurface>
<gml:MultiSurface srsName=“EPSG:4166” srsDimension=“3”>
<gml:surfaceMember>>
…
<gml:LinearRing>
<gml:posList>−1745.7374937091952 202948.24544274173 −4260 −1745.7374937091952 202948.24544274173 −4280 −1734.0178178638214 202926.1626621632 −4280 −1734.0178178638214 202926.1626621632 −4300 −1776.2086509071673 203005.6606722459 −4300 −1776.2086509071673 203005.6606722459 −4280 −1764.4889750617936 202983.57789166737 −4280 −1764.4889750617936 202983.57789166737 −4260 −1745.7374937091952 202948.24544274173 −4260</gml:posList>
</gml:LinearRing>
…
</bldg:Door>
</core:cityObjectMember>

**Table 3:** Comparison between file types and file sizes.

Model	Revit 2023	IFC	CityGML LOD4	3D Tiles
Community Center	99,684 KB	17,824 KB	310,055 KB	329,610 KB

## Data Availability

No new data were created or analyzed in this study. Data sharing is not applicable to this article.
